# Book Reviews

**Published:** 1898-08

**Authors:** 


### Book Reviews.

MANUAL OF PHYSICAL DIAGNOSIS, FOR THE USE OF STUDENTS AND PHYSICIANS. By James Tyson, M.D. Philadelphia: P. Blakiston, Son & Co. 1898.

The third edition of this work contains, besides the usual subjects considered in this connection, an enlarged section on blood examination, counting corpuscles, measuring hemoglobin, preparation of stained films. There are also instructions for examining sputum, with an account of the several methods. There is a chapter on analysis of stomach contents, with directions how to make and use the various reagents employed; a short illustrated notice of the Roentgen Ray in diagnosis; clear directions for making an autopsy for clinical purposes. The latter is not generally included in a work on physical diagnosis, but its insertion will give the purchaser of the book all information necessary for a complete study of the subject of disease and it is therefore not out of place. The little volume is profusely illustrated.

HAND BOOK OF MATERIA MEDICA FOR TRAINED NURSES, including sections in Therapeutics and Toxicology and a Glossary of Terms, with dose and use of each drug. By John E. Groff, Ph. G., Apothecary in the Rhode Island Hospital, Providence. Price $1.25. Philadelphia: P. Blakiston, Son & Co. 1898.

In this book of 227 pages the author has presented the subject of Materia Medica in the form of an abridgment especially adapted to the needs of trained nurses. It is desirable that nurses should have some familiarity with the various drugs used in the treatment of disease and their appropriate dose. The subject is discussed under the following headings: "Weights and Measures," "Crude Drugs," "Preparations," "Dosage Under Chemistry Elements," "Acids," "Alkalies," "Metals," "Reconsideration," "Organic Chemistry," "Fermentation Products," "Volatile Oils," "Fixed Oils and Fats," "Glucosides and Alkaloids," "Drugs from the Animal Kingdom," "The Thermometer."

In an appendix there are short chapters on "Therapeutics," "Toxicology," and finally a "Glossary." The work is replete with practical rules which will be found of great use. At the end of each chapter there is a list of questions reviewing the ground covered in the text. The book is one of practical value and can be recommended to nurses. It also makes a handy reference volume for the doctor's library.

RELIGION AND LUST. By James Weir, M.D., Owensboro, Ky.

This book takes its name from the largest essay contained in it. There are others on social and general subjects.

In the first of this collection the author endeavors to show that ecclesiastic forms and religious ceremonial are closely allied to, and are sprung from, the ancient worship of the god Priapus under his symbol, the phallus.

The argument is by no means conclusive. The other articles are interesting compilations of facts culled from various sources, such as prostitution in middle ages, Moll's and Kraft-Ebing's works on sexual perversion, writers in the archives d'antropologie criminelle, and the like. The French and Italians have worked over the field of sociology and criminal anthropology so thoroughly that there remains very little to be said that is new or true. Dr. Weir is well informed on these topics, and the English reader, if he has a similar taste, will find much to interest him in this volume.

---

Laboratory Text-book of Pathology.—For the use of students and practitioners of medicine. By Horace J. Whitacre, B.S., M.D., Cincinnati. Price, $1.50. Philadelphia: P. Blakiston, Son & Co. 1898.

This book is designed as a working manual for the student in microscopy; one that he may have at his side for reference in identifying the objects of study. It is illustrated with photomicrographs and accurate drawings, and is very clear in the text. The work seems admirable adapted for the purposes set forth by its author.

---

Conservative Gynecology and Electro-Therapeutics, a practical treatise on the diseases of women and their treatment by electricity, third edition, revised, rewritten and greatly enlarged. By G. Betton Massey, M.D., Physician to the Gynecic Department of Howard Hospital, Philadelphia, late Electro-Therapeutist to the Infirmary for Nervous Diseases, Philadelphia, Fellow and ex-President of the American Electro-Therapeutic Association, of the Societe Francaise d'Electro-therapie, of the American Medical Association, etc. Illustrated with twelve full-page original chromo-lithographic plates in twelve colors, numerous full-page original half-tone plates of photographs taken from nature, and many other engravings in the text. Royal octavo, 400 pages. Extra cloth, beveled edges, $3.50 net. The F. A. Davis Co., Publishers, 1914-16 Cherry street, Philadelphia, 117 West Forty-second street, New York City, 9 Lakeside Building, 218-220 South Clark street, Chicago, Ill.

The author is well known from his "Electricity in the Diseases of Women" and his frequent contributions to the literature of electro-therapeutics as applied to gynecology. In the present volume he gives his experience and observations on the use of electricity in pelvic disorders and fully substantiates through clinical evidence the truth of his statements. The work contains a section on electricity generally considered. It is profusely illustrated with cuts and photographs after the usual excellent manner of the Davis publications.

---

A Compend of Diseases of the Skin. By Dr. Jay F. Schamberg, associate in skin diseases of the Philadelphia Polyclinic. Philadelphia: P. Blakiston, Son & Co. 1898.

This is an excellent little book and is destined to prove of great use to the student as an aid to the study of dermatology. Only known facts are included in the consideration of the 135 diseased conditions of the skin discussed in the book. The subject is well illustrated. Duhring's classification is the one adopted.

